# Lower serum magnesium concentration is associated with diabetes, insulin resistance, and obesity in South Asian and white Canadian women but not men

**DOI:** 10.3402/fnr.v59.25974

**Published:** 2015-05-05

**Authors:** Jesse Bertinato, Chao Wu Xiao, W. M. Nimal Ratnayake, Lois Fernandez, Christopher Lavergne, Carla Wood, Eleonora Swist

**Affiliations:** 1 Nutrition Research Division, Health Products and Food Branch, Health Canada, Ottawa, Canada; 2 Department of Biochemistry, Microbiology and Immunology, University of Ottawa, Ottawa, Canada; 3 Department of Cellular and Molecular Medicine, University of Ottawa, Ottawa, Canada; 4 Department of Biology, University of Ottawa, Ottawa, Canada

**Keywords:** adults, body mass index, glucose, homeostatic model assessment of insulin resistance, magnesium status, McAuley's index, quantitative insulin sensitivity check index

## Abstract

**Background:**

A large proportion of adults in North America are not meeting recommended intakes for magnesium (Mg). Women and people of South Asian race may be at higher risk for Mg deficiency because of lower Mg intakes relative to requirements and increased susceptibility to diabetes, respectively.

**Objective:**

This study compared serum Mg concentrations in South Asian (*n=*276) and white (*n=*315) Canadian women and men aged 20–79 years living in Canada's Capital Region and examined the relationship with diabetes, glucose control, insulin resistance, and body mass index.

**Results:**

Serum Mg concentration was lower in women of both races and South Asians of both genders. Racial differences in serum Mg were not significant after controlling for use of diabetes medication. A substantial proportion of South Asian (18%) and white (9%) women had serum Mg <0.75 mmol/L indicating hypomagnesemia. Use of diabetes medication and indicators of poorer glucose control, insulin resistance, and obesity were associated with lower serum Mg in women, but not in men.

**Conclusions:**

These results suggest that the higher incidence of diabetes in South Asians increases their risk for Mg deficiency and that health conditions that increase Mg requirements have a greater effect on Mg status in women than men.

Magnesium (Mg) is an essential co-factor required for many biochemical reactions and plays an important role in glucose metabolism (1). There is evidence that higher Mg intakes and serum Mg concentrations may decrease the risk of insulin resistance and type 2 diabetes (2–7). It is also well established that diabetes predisposes individuals to Mg deficiency. Greater loss of Mg in urine secondary to glycosuria and insulin resistance as well as other factors have been proposed to explain this effect (8).

Nutrition surveys show that a large proportion of adults in North America have inadequate Mg intakes (9, 10). The Canadian Community Health Survey 2.2 (2004) revealed that more than 34% of adults over the age of 19 years had Mg intakes from food below the estimated average requirement (EAR), with percentages greater than 40% in several age and sex groups (10). These data suggest a possible problem of widespread Mg deficiency in Canada, yet to date the physiological Mg status of a similar nationally representative Canadian cohort has not been determined.

Some subpopulations may be at greater risk for Mg deficiency. South Asians make up the largest visible minority group in Canada, accounting for 4.8% of the total population in 2011 (11). South Asians are more susceptible to major chronic diseases such as cardiovascular disease and diabetes compared to whites of European descent and other races (12–14). The higher prevalence of diabetes in South Asians may increase their risk for Mg deficiency.

Women may also be at greater risk for Mg deficiency. Mg requirements for the North American population were set substantially higher for men than for women. For adults aged 31–50 years, the EAR for men and women is 350 and 265 mg/day, respectively (15). More recent data from balance studies have suggested that Mg requirements for healthy women and men may be similar (16). Similar Mg requirements for both genders would put women at greater risk for Mg deficiency because women have lower Mg intakes (9).

Total serum Mg concentration is an established biomarker of Mg status (17). The reference interval was determined to be 0.75–0.955 mmol/L in a US adult population aged 18–74 years (18). Although there is some debate over what the lower cut-off value should be, a serum Mg concentration <0.75 mmol/L is generally accepted as indicating hypomagnesemia (suggesting Mg deficiency) (19, 20). Individuals with a serum Mg value of 0.75 –<0.85 mmol/L have been said to have chronic latent Mg deficiency (CLMD), which has been described as having a small chronic negative Mg balance with serum Mg concentration within the lower part of the reference interval (19, 20).

Presently there is little information on the prevalence of Mg deficiency in Canadians, including in potentially vulnerable subpopulations. The objectives of this study were to compare serum Mg concentrations of South Asian and white Canadian women and men and to examine the relationship with diabetes, body mass index (BMI), and measures of glucose control and insulin resistance.

## Materials and methods

### Participants and study protocol

This study reports results from an observational study entitled ‘Assessment of vitamin D, omega-3 and blood lipid risk factors for cardiovascular disease in South Asian and white Canadians living in Ottawa’. The study protocol was approved by the Health Canada and Public Health Agency of Canada Research Ethics Board (Protocol No.: 2010-0043). All participants provided written informed consent. South Asian and white Canadian adults were recruited from Canada's Capital Region through local advertisements. Blood samples were drawn from the same participants at two seasonal time periods: April–May 2012 (after winter) and September–October 2012 (after summer). This study reports results from the September–October time period (South Asians, *n=*276; whites, *n=*315). At the time of blood collection, participants’ height and weight (in light clothing without shoes) were measured using a Seca column scale (Hamburg, Germany) with automatic BMI calculation (weight in kilograms divided by height in meters squared). Prior to attending the clinic, participants completed a demographic questionnaire to obtain information on date of birth, gender, and race. For assessment of race, participants were asked to identify themselves as South Asian (e.g. Indian, Sri Lankan, Pakistani, Bangladeshi, or Nepalese), white of European descent, or other race. All participants were also asked to complete a health questionnaire to identify participants taking certain medications and having specific chronic diseases. Women and men aged 20–79 years of South Asian or white European descent were eligible to participate in the study. People of other ages or races were excluded. There were no other exclusion criteria.

### Blood collection and processing

Venous blood samples were collected following an overnight fast (>10 h). Blood was collected in BD Vacutainer Plus™ serum tubes (Cat. No.: 02-685-111, Thermo Fisher Scientific, Waltham, MA, USA) and BD Vacutainer™ tubes with K_2_EDTA (Cat. No.: 02-683-99B, Thermo Fisher Scientific) for isolation of serum and plasma, respectively. Blood tubes were centrifuged (2280×g, 30 min) and serum and plasma were collected and frozen in aliquots at −80°C until analysis.

### Assays and calculations

Total serum Mg, triglycerides, and glucose were measured with the Ortho Clinical Diagnostics Vitros 5,1 FS analyser (Johnson and Johnson, Piscataway, NJ, USA). Results from serum samples with level of hemolysis exceeding the threshold value for the assay were excluded from the analyses. Insulin was measured using an insulin ELISA (Cat. No.: 80-INSHU-E01.1, E10.1, ALPCO, Salem, NH, USA). Insulin resistance was assessed using fasting plasma insulin; quantitative insulin sensitivity check index (QUICKI), 1/[log insulin µIU/mL)+log glucose (mg/dL)]; homeostatic model assessment of insulin resistance (HOMA-IR), [glucose (mmol/L)×insulin (µIU/mL)]/22.5; and McAuley's index, exp[2.63 – 0.28 ln insulin (µIU/mL) – 0.31 ln triglycerides (mmol/L)]. Established cut-off values for impaired fasting glucose (indicating pre-diabetes) and insulin resistance were used for the analyses: fasting serum glucose, ≥5.6 mmol/L (21); fasting plasma insulin, ≥12 µIU/mL; QUICKI, ≤0.33; HOMA-IR, ≥2.6; and McAuley's index, ≤5.8 (22–25).

### Statistical analyses

Data were reported as percentages or means±SEM. Differences between means were determined by one-way ANOVA. When overall results were significant, Fisher's least significant difference test was used to identify which groups differed. When the data were not normally distributed or group variances were unequal, the data were transformed and reanalyzed. If normality and equality of variances could not be achieved, the non-parametric Kruskal–Wallis ANOVA was used to determine differences among groups. Differences in proportions were determined by chi-square test or Fisher's exact test. Statistical significance was set at *p*<0.05. Data were analyzed using Statistica 12 (StatSoft, Tulsa, OK, USA) and SigmaPlot 11.2 (Systat Software, Inc., San Jose, CA, USA).

## Results

**Fig. 1:**
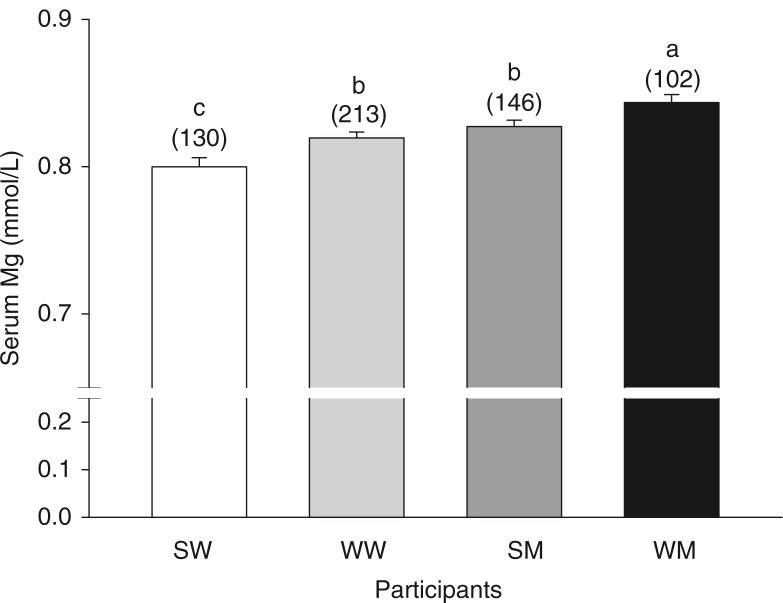
Serum Mg concentrations of participants. Bars represent the means±SEM. Numbers above the bars in parentheses indicate the number of participants in that group. Bars without a common letter differ, *p<*0.05. SM, South Asian men; SW, South Asian women; WM, white men; WW, white women.

The serum Mg concentrations of women and men of South Asian and white European descent were compared (Fig. 1). Women had lower serum Mg compared to men of the same race. South Asians had lower serum Mg than whites of the same gender. The mean ages of the South Asian women (SW), white women (WW), South Asian men (SM), and white men (WM) were 46±1, 47±1, 48±1, and 49±1 years, respectively. Age did not differ (*p*≥0.05) between groups.

**Fig. 2:**
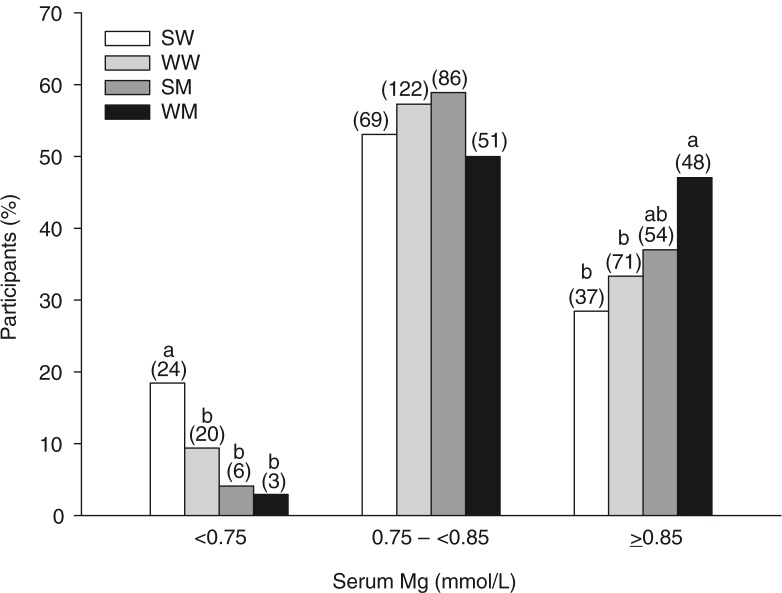
Classification of participants according to serum Mg concentrations. Bars represent the percentages of participants with hypomagnesemia (<0.75 mmol/L), chronic latent Mg deficiency (0.75 –<0.85 mmol/L), or normal serum Mg (≥0.85 mmol/L). Numbers above the bars in parentheses indicate the number of participants in that group. Bars without a common letter within each category differ, *p<*0.05. SM, South Asian men; SW, South Asian women; WM, white men; WW, white women.

To examine the prevalence of Mg deficiency in our South Asian and white cohorts, participants were categorized as having hypomagnesemia (<0.75 mmol/L), CLMD (0.75 –<0.85 mmol/L), or normal serum Mg (≥0.85 mmol/L). The proportion of SW (18%) with serum Mg <0.75 mmol/L was higher compared to WW (9%), SM (4%), and WM (3%) (Fig. 2). The proportion of SW and WW with serum Mg ≥0.85 mmol/L was lower compared to WM.

**Fig. 3:**
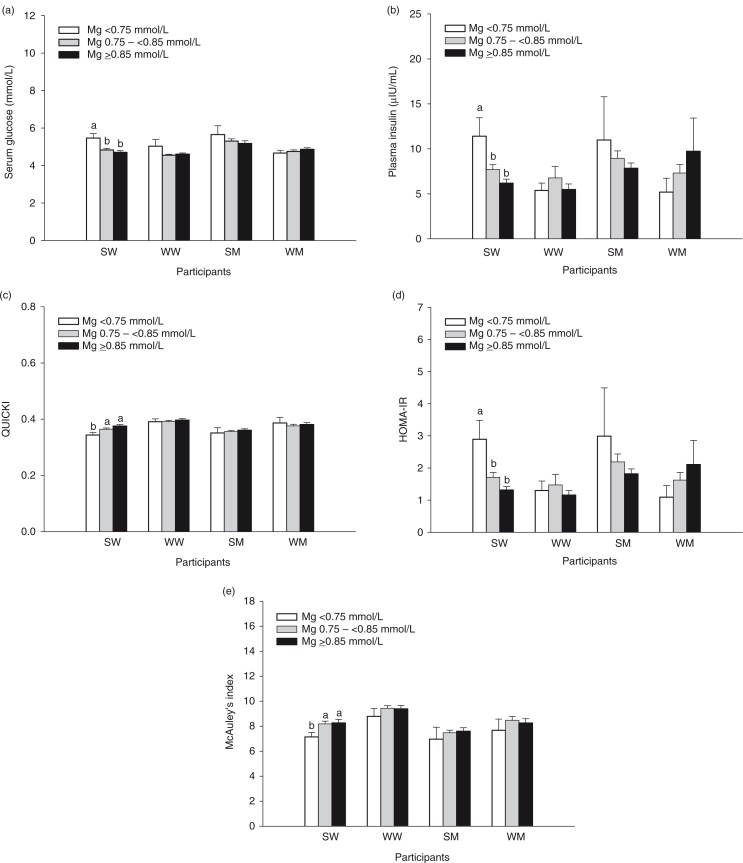
Fasting serum glucose (a), fasting plasma insulin (b), QUICKI (c), HOMA-IR (d), and McAuley's index (e) of participants categorized as having hypomagnesemia (<0.75 mmol/L), chronic latent Mg deficiency (0.75 –<0.85 mmol/L), or normal serum Mg (≥0.85 mmol/L). Bars represent the means±SEM. Number of participants in each group in panels (a–e) are the same as shown in Fig. 2. Bars without a common letter for each group of participants differ, *p<*0.05. SM, South Asian men; SW, South Asian women; WM, white men; WW, white women.

The relationship between serum Mg concentration and indicators of glucose control and insulin resistance was examined. SW with serum Mg <0.75 mmol/L had higher serum glucose, higher plasma insulin, lower QUICKI, higher HOMA-IR, and lower McAuley's index compared to SW with serum Mg between 0.75 and <0.85 mmol/L or ≥0.85 mmol/L (Fig. 3a–e). None of the indicators differed between WW or men categorized in different groups according to serum Mg concentration (Fig. 3a–e).

**Fig. 4:**
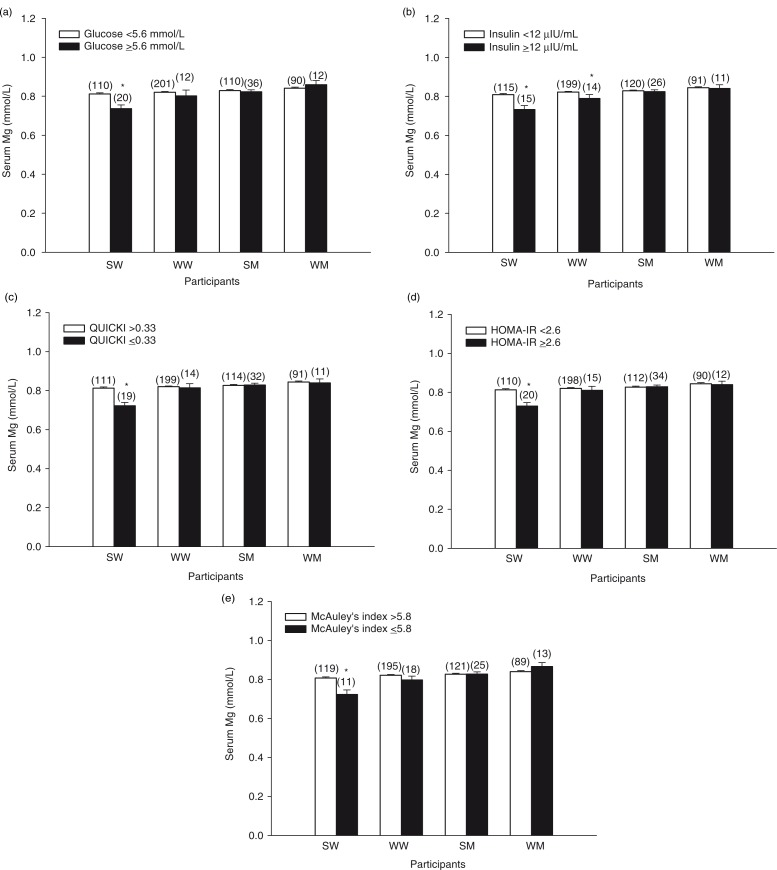
Serum Mg concentrations of participants categorized as having normal (white bars) or abnormal (black bars) values for fasting serum glucose (a), fasting plasma insulin (b), QUICKI (c), HOMA-IR (d) and McAuley's index (e) based on cut-off values for impaired glucose control and insulin resistance. Bars represent the means±SEM. Numbers above the bars in parentheses indicate the number of participants in that group. *Different from participants with normal values, *p<*0.05. SM, South Asian men; SW, South Asian women; WM, white men; WW, white women.

Serum Mg concentrations were compared in participants categorized as having normal or abnormal values for fasting glucose and indicators of insulin resistance based on established cut-off values. SW with abnormal values for serum glucose (≥5.6 mmol/L), plasma insulin (≥12 µIU/mL), QUICKI (≤0.33), HOMA-IR (≥2.6), or McAuley's index (≤5.8) had lower serum Mg than SW with normal values (Fig. 4a–e). WW with abnormal plasma insulin had lower serum Mg (Fig. 4b). Serum Mg concentration did not differ between men with normal or abnormal values (Fig. 4a–e).

**Fig. 5:**
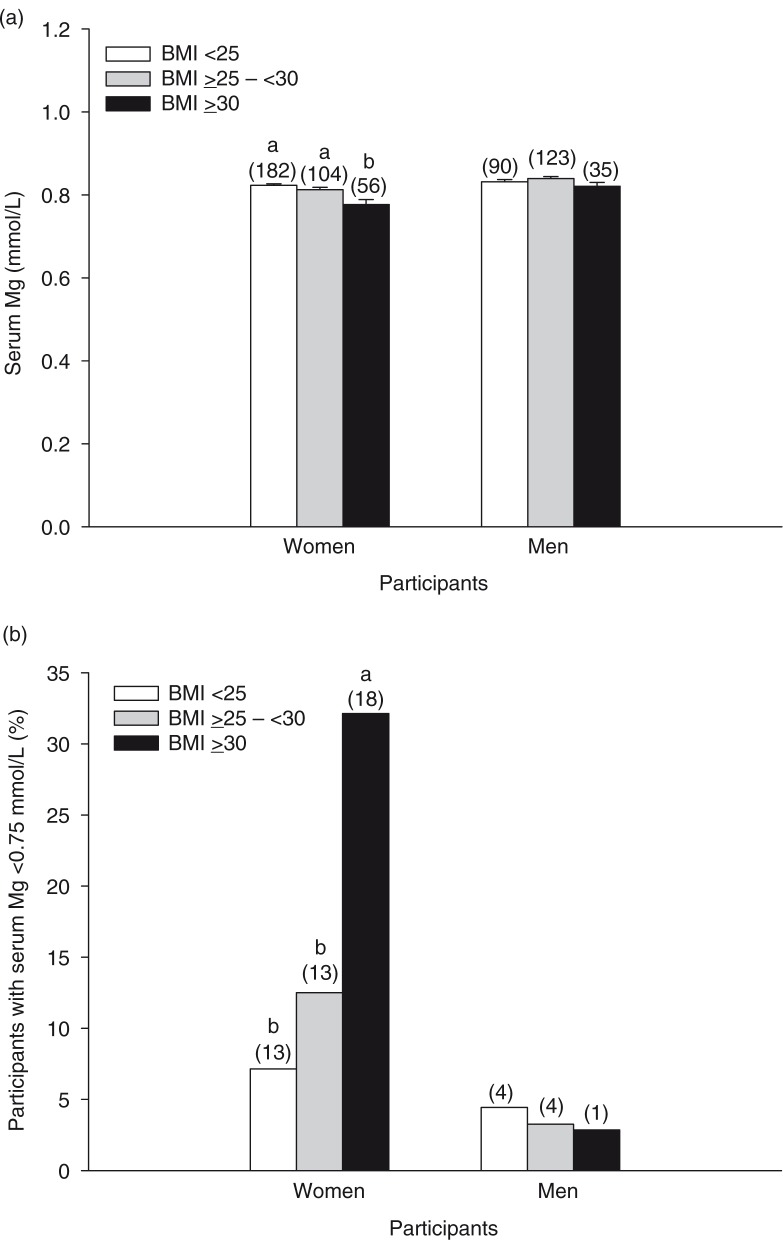
Serum Mg concentrations of women and men with a BMI <25 (normal weight), ≥25 –<30 (overweight), or ≥30 (obese) (a). Bars represent the means±SEM. Percentages of women and men with hypomagnesemia according to BMI categories (b). Numbers above the bars in parentheses indicate the number of participants in that group. Bars without a common letter differ, *p<*0.05.

The relationship between BMI and serum Mg concentration was investigated. The participants were categorized as normal weight (BMI <25), overweight (BMI ≥25 –<30) or obese (BMI ≥30) based on BMI cut-off values established by the World Health Organization (WHO) (26). The effect of BMI on serum Mg was modified by gender (*p=*0.04), but not race (*p*≥0.05). Thus, the data are presented for both races combined. Obese women had lower serum Mg compared to normal weight or overweight women (Fig. 5a). Serum Mg did not differ between men categorized by BMI (Fig. 5a). The percentage of obese women with a serum Mg concentration <0.75 mmol/L was higher compared to normal weight or overweight women (Fig. 5b).

A WHO expert consultation concluded that Asians generally have higher percentage body fat than whites of the same BMI (27). Therefore, we reanalyzed the data using lower BMI cut-off points for South Asians as suggested by the WHO (27): normal weight (BMI <23), overweight (BMI ≥23 –<27.5), and obese (BMI ≥27.5). Obese women had lower (*p*<0.05) serum Mg (0.78±0.01 mmol/L, *n*=76) compared to normal (0.82±0.00 mmol/L, *n=*154) or overweight (0.82±0.01 mmol/L, *n=*112) women. Differences were not observed (*p*≥0.05) between normal (0.84±0.01 mmol/L, *n=*64), overweight (0.84±0.00 mmol/L, *n=*120), or obese (0.82±0.01 mmol/L, *n=*64) men. A higher percentage (*p*<0.05) of obese women had hypomagnesemia (28.9%, *n*=22) compared to normal (7.8%, *n=*12) or overweight (8.9%, *n=*10) women. Percentages of men with hypomagnesemia did not differ (*p*≥0.05) among normal (3.1%, *n=*2), overweight (3.3%, *n=*4) and obese (4.7%, *n=*3) groups.

In total, 98% of participants completed a health questionnaire providing information on use of certain medications and specific chronic diseases. A larger proportion of WW reported not taking medication for diabetes compared to SW and SM (Table 1). After accounting for use of diabetes medication, the effect of race on serum Mg was not significant (Table 1). Because gender modified the effect of diabetes medication use on serum Mg, women and men were analyzed separately. Race did not affect (*p*≥0.05) serum Mg after controlling for diabetes medication use in both women and men. Mean serum Mg was markedly lower for women that reported using diabetes medication compared to non-users (0.70±0.02 vs. 0.82±0.00 mmol/L, *p<*0.0001). In contrast, mean serum Mg for men that reported using diabetes medication was not significantly lower than non-users (0.80±0.01 vs. 0.84±0.00 mmol/L, *p=*0.07). Eighty percent of women that reported using medication for diabetes had serum Mg <0.75 mmol/L, compared to only 12% of men (*p<*0.05). Use of other medications or having specific chronic diseases did not affect serum Mg (Table 1).

**Table 1:** Effect of medication use and chronic diseases on serum Mg concentration

Characteristic	Participants (*n*)^3^				ANOVA *p*-value^4^				
	
	SW	WW	SM	WM	Characteristic	Race	Gender	Characteristic×race	Characteristic×gender
DM					<0.0001	NS	<0.0001	NS	0.0002
No	113^b^	208^a^	126^b^	98^ab^					
Yes	11	4	14	3					
OM^1^					NS	0.0002	<0.0001	NS	NS
No	103	173	102	81					
Yes	21	39	38	20					
CD^2^					NS	0.01	0.001	NS	NS
No	115	201	128	91					
Yes	9	11	12	10					

1 Taking one or more of the following medications: amlodipine/atorvastatin, atorvastatin, calcium channel blockers, cholestyramine, cimetidine, colesevelam, colestipol, corticosteroids, estrogens, ezetimibe/simvastatin, fenofibrate, fluvastatin, gemfibrozil, heparin, isoniazid, ketoconazole, lovastatin, neomycin, niacin, niacin/lovastatin, orlistat, raloxifene, rifampin, rosuvastatin, simvastatin, thiazide diuretics, thioridazine, verapamil, or cholesterol-lowering medication.

2 Having one or more of the following diseases: celiac disease, chronic kidney disease, Crohn's disease, cystic fibrosis, heart disease, hyperthyroidism, liver dysfunction, primary hyperparathyroidism, tumor-induced osteomalacia, or Whipple's disease.

3 Values in a row without a common letter signify differences in the proportions of participants with that characteristic, *p<*0.05.

4 Data were analyzed using multifactorial ANOVA to test for effects and interactions of the independent variables (characteristic, race and gender) on serum Mg (dependent variable). NS, *p*≥0.05. CD, chronic disease; DM, diabetes medication; NS, not significant; OM, other medication; SM, South Asian men; SW, South Asian women; WM, white men; WW, white women.

## Discussion

Canada's Capital Region has a diverse South Asian community with a large population of nearly 34,000. Whites of European descent make up the largest racial group in this region, accounting for almost three-quarters of the population. Because the only inclusion criteria for this study were South Asian or white European descent and 20–79 years of age, it is likely that participants closely represent the general adult population of these two races living in this area.

Gender and racial differences in serum Mg concentrations were observed. Although serum Mg has been reported to vary with age (18), age is an unlikely explanation for the observed differences. The mean ages of women and men of both races did not differ.

Serum Mg was lower in women than men, regardless of race. Furthermore, a larger percentage of SW compared to SM had hypomagnesemia, suggesting that women are at greater risk for Mg deficiency. Mg requirements for the North American population were set substantially higher for men than for women (15). More recent research has suggested that Mg requirements may be lower than previously estimated (EAR=165 mg/day) and similar for women and men (16). The lower serum Mg and higher incidence of hypomagnesemia in women in this study may be explained by the lower Mg intakes for women relative to requirements. A limitation of the present study is that information on Mg intake from food and supplements was not collected. However, Mg intake follows caloric intake and therefore would be expected to be lower for women. In a national US survey, lower Mg intakes were reported for women of white European, African, and Mexican descent (9).

Racial differences in serum Mg exist. In a multi-racial cohort of adults living in New York City, Hispanics had lower serum Mg concentrations compared to African Americans (28). White Americans were reported to have higher serum Mg than black Americans (18, 29). Differences between whites and blacks were observed in both genders and across many age groups (18). Black Americans were shown to have lower Mg intakes compared to white Americans, which may explain, at least in part, the lower serum Mg (9, 29). In this study, serum Mg was lower in South Asians compared to whites in both genders. However, after accounting for use of diabetes medication, the racial differences were no longer significant, suggesting that diabetes and/or diabetes medication use was a main factor accounting for the lower serum Mg observed for South Asians. It is important to mention that of the 32 subjects that reported taking diabetes medication, only 6 subjects (2 women and 4 men) had well-controlled fasting glucose levels (i.e. <5.6 mmol/L).

The hyperinsulinemic-euglycemic clamp is considered the gold standard for evaluation of insulin sensitivity, yet the complexity of the procedure has led to development of more practical, indirect methods for use in larger epidemiological studies, such as QUICKI (30), HOMA-IR (31, 32), and McAuley's index (31, 32). QUICKI and HOMA-IR are based on glucose and insulin measurements, whereas McAuley's index is an insulin- and triglyceride-based method. QUICKI and HOMA-IR have been shown to be valid surrogate indicators of insulin sensitivity in South Asians (33). SW with serum Mg concentrations <0.75 mmol/L showed differences in serum glucose, plasma insulin, QUICKI, HOMA-IR, and McAuley's index, indicating poorer glucose control and greater insulin resistance. In addition, SW with abnormal values for these measures had lower serum Mg, corroborating the association between lower serum Mg and poorer glucose control and insulin resistance. Notably, with regard to measures of glucose control and insulin resistance examined in this study, participants with serum Mg of 0.75 –<0.85 mmol/L were indistinguishable from participants with serum Mg ≥0.85 mmol/L.

A larger number of SM compared to SW had abnormal values for fasting glucose and indicators of insulin resistance, yet serum Mg was not lower in SM with abnormal values. In women, serum Mg was markedly lower for users of diabetes medication and a large proportion of women using diabetes medication were hypomagnesemic. In contrast, a much smaller proportion of men using diabetes medication were hypomagnesemic and serum Mg was not significantly lower in men using diabetes medication compared to non-users. These results suggest that women are more vulnerable to reductions in Mg status caused by factors that increase magnesium requirements, such as insulin resistance and diabetes. Given the important role of Mg in insulin action (8, 34), it is also possible that the higher prevalence of hypomagnesemia in women contributed to poorer glucose control and insulin resistance rather than just being a consequence of the abnormalities.

Obesity is a major problem in Canada, with approximately a quarter of Canadian adults considered obese (35). Presently, the relationship between obesity and Mg status is unclear. Studies have shown that overweight/obese children have lower serum Mg compared to lean children (5, 36). A study in Canadian adults from Newfoundland and Labrador showed that Mg intake was more strongly associated with insulin resistance in overweight and obese participants (4). In the present study, obese women had lower serum Mg compared to normal weight or overweight women. Furthermore, a larger percentage of obese women were hypomagnesemic. Serum Mg did not differ among BMI groups for men, suggesting that obesity is more detrimental to Mg status for women. Results were similar when using the same BMI cut-off points for whites and South Asians or when using lower cut-off points for South Asians, given evidence suggesting that lower BMI cut-off points for some Asian populations may better estimate obesity-related health risks (27). Obesity is a risk factor for type 2 diabetes and insulin resistance and therefore can affect Mg status by altering glucose metabolism and insulin action. However, obesity may also affect Mg status by other mechanisms. Further research exploring the effect of obesity on Mg metabolism and requirements in both genders will be important.

Serum Mg has limitations as a biomarker of Mg status. Because only a small fraction (<1%) of total body Mg is found in the serum, serum Mg may not always accurately reflect intracellular Mg status. However, low serum Mg is usually indicative of a systemic Mg deficiency. Serum Mg is also influenced by several factors including renal function and albumin concentrations. Serum Mg is linearly related to albumin concentrations at high and low albumin concentrations (37); therefore, hypoalbuminemic conditions may lead to spuriously low serum Mg concentrations. It is unconventional, however, to adjust serum Mg for albumin concentrations and only one subject in this study reported having chronic kidney disease.

In this study, gender and racial differences in serum Mg concentrations were observed in adults living in Canada's Capital Region. The results suggest that South Asians and women are two vulnerable subpopulations for Mg deficiency. The higher occurrence of diabetes in South Asians likely increases their risk for Mg deficiency. The association of lower serum Mg with diabetes, insulin resistance, and obesity only in women suggests greater vulnerability of women to reductions in Mg status by factors that increase Mg requirements. This increased vulnerability may be explained by lower Mg intakes relative to nutritional needs. Given that a substantial proportion of women were found to be hypomagnesemic, further research evaluating the prevalence of Mg deficiency in the Canadian population is needed, including in minority racial groups that may not be captured in national-level surveys. This research will help to assess the health implications of the observation that many Canadians are not meeting the current recommended intakes for Mg.
